# Psychological and Physical Intimate Partner Violence, Measured by the New York City Community Health Survey — New York City, 2018

**DOI:** 10.1007/s10896-022-00442-1

**Published:** 2022-09-26

**Authors:** Karen A. Alroy, Amy Wang, Michael Sanderson, L. Hannah Gould, Catherine Stayton

**Affiliations:** 1 Epidemic Intelligence Service, Division of Scientific Education and Professional Development, Centers for Disease Control and Prevention, Atlanta, GA USA; 2 Bureau of Epidemiology Services, Division of Epidemiology, New York City Department of Health and Mental Hygiene, New York City, NY USA; 3 Bureau of Environmental Disease and Injury Prevention, Division of Environmental Health, New York City Department of Health and Mental Hygiene, New York City, NY USA

**Keywords:** Intimate partner violence, IPV, Psychological aggression, Physical intimate partner violence, Abuse, New York City, Population health

## Abstract

**Purpose:**

Intimate partner violence (IPV) can damage long-term physical and mental health, yet IPV prevalence in New York City (NYC) is unknown. We described prevalence and health correlates of psychological and physical IPV in NYC.

**Method:**

The 2018 NYC Community Health Survey, a representative telephone survey among adult residents, asked about lifetime psychological or physical IPV experiences. We estimated age-adjusted physical and psychological prevalence, stratified by demographic variables, and created log-linear multivariable models with 95% CIs to measure the association of each IPV type with health conditions and behaviors.

**Results:**

Overall, 10,076 surveys were completed. We excluded responses with missing IPV values. Of 9,945 adults, 16.7% reported ever having experienced psychological IPV; higher prevalence among females (18.6%; CI:17.0–20.2) than males (14.5%; CI:13.1–16.2). Prevalence of not getting needed mental health treatment (PR: 4.5; CI:3.3–6.1) and current depression (PR:2.6 CI:2.1–3.1) was higher among adults who had ever experienced psychological IPV, compared with those who had not. Of 9,964 adults, 9.8% reported ever having experienced physical IPV; higher prevalence among females (12.4%; CI:11.1–13.8) than males (6.8%; CI:5.8–8.0). Prevalence of not getting needed mental health treatment (PR:3.9, CI:2.8–5.4) and current depression (PR:2.6, CI:2.1–3.2) was higher among adults who had ever experienced physical IPV, compared with those who had not.

**Conclusions:**

One in six (16.7%) and one in 10 (9.8%) NYC adults reported ever experiencing psychological IPV and ever experiencing physical IPV, respectively. Key implications suggest that IPV potentially underlies public health priority health conditions and behaviors.

**Supplementary Information:**

The online version contains supplementary material available at 10.1007/s10896-022-00442-1.

## Introduction

Intimate partner violence (IPV) encompasses a variety of aggressive psychological and physical behaviors that can occur between partners in a current or prior relationship (Breiding et al., 2015). IPV can result in both acute and chronic impacts to health (Basile et al., 2004; Black, 2011; Campbell, 2002; Petrosky et al., 2017; Plichta, 2004; Smith et al., 2017). Exposure to IPV can accumulate over a lifetime, complicating measurement of IPV prevalence and quantification of health conditions and behaviors associated with IPV.

To measure IPV, certain jurisdictions have used law enforcement data, violent injury surveillance, or calls to hotlines, family shelters and other domestic violence service providers, such as the Family Justice Centers located in each of the five boroughs of New York City (NYC) (NYC Mayor’s Office to End Domestic & Gender-Based Violence, 2021; Pattavina et al., 2007; Stayton et al., 2008). However, these data sources alone might be inadequate to accurately estimate IPV burden (Zepp, 1996). Some people might fear police involvement and be reluctant to contact law enforcement when intimate partner disputes turn violent (Decker et al., 2019; Wolf et al., 2003). Not all people who experience IPV reach out for support from hotlines or service providers. Complex obstacles can prevent people from seeking help and wanting to have their IPV experiences documented. Systemic racism, oppression, and fear can be at the root of why people are reluctant to reach out for help (Decker et al., 2019, Hampton et al., 2003; Earner, 2010; Kim & Sung, 2016; Muchow & Amuedo-Dorantes, 2020). Calls to law enforcement for IPV are typically associated with the most severe forms of violence including threats with a weapon, injury, and the destruction of property (Akers & Kaukinen, 2009). Consequently, police or service provider calls only measure the acute nature of the most violent episodes and do not describe the accumulation or escalation of violence that is typical of IPV. The insidious and subtle injuries that can result from IPV, such as headache, anxiety, chronic fatigue, and gastrointestinal disturbances, are not typically detectable through violent injury surveillance systems (Black, 2011).

We used 2018 data from an annual cross-sectional, population-based, general health survey to assess lifetime prevalence estimates, demographics, and health correlates of physical IPV and psychological IPV among NYC adults. This 2018 survey was the first-time questions on both lifetime psychological IPV (2018) and lifetime physical IPV (2016 & 2018) were asked in a representative citywide NYC survey. This work fills an important gap in the literature because there are no current population-based estimates of IPV prevalence or IPV-related health correlates among NYC residents by both types of IPV. Population-based surveys offer an approach that can increase representativeness and accuracy of prevalence estimates (Breiding et al., 2015; Rhodes et al., 2002). Although an IPV-focused survey could offer valuable and comprehensive information, implementing a new survey can be cost prohibitive for local health jurisdictions. In this case, IPV related questions were added to an existing general health survey. While there are many IPV types, we only had space to include two questions about psychological and physical IPV and were unable to ask about sexual violence, stalking, or other IPV types. By asking questions on lifetime exposures, our measurement approach captured experiences that could happen at any time in life, therefore including more than just the most extreme or severe instances of IPV. This is important since for many people IPV can occur repeatedly over a lifetime.

An additional strength of collecting lifetime IPV data with a general health survey is that general health surveys collect wide-ranging self-reported physical health and health behavior data (NYC DOHMH, 2020). The broad question distribution in the general health survey allowed for analyses of a number of health correlates of IPV. We examined health conditions and behaviors including access to care, depression, alcohol use, tobacco use, self-reported health, and hypertension because prior literature have correlated these conditions and behaviors with IPV, they have mechanistic plausibility, and have been identified as priority public health issues by the NYC Department of Health and Mental Hygiene (DOHMH) (Black, 2011, Wong et al., 2011, Lipsky & Caetano, 2007, Creech et al., 2012, Cerulli et al., 2012, Campbell, 2002, Beydoun et al., 2012, Basile et al., 2004, Mettey et al., 2015). Thus, population-based survey questions such as these can collect essential adjunct data, complementing limitations of other service-oriented data sources.

We conducted our analysis on data from the NYC Community Health Survey (CHS) to estimate the prevalence of self-reported IPV among NYC adults, to clarify which populations in NYC have experienced IPV, and to identify and measure associated health conditions and behaviors. Studies on IPV have reported that females are more likely than males to experience IPV, and to be injured or killed by an intimate partner (Black, 2011; Petrosky et al., 2017). We hypothesized higher IPV prevalence among females, as well as higher among other populations that experience discrimination and exclusion such lesbian, gay, and bisexual (LGB) adults for example (Walters et al., 2013; Edwards et al., 2015; Brown & Herman, 2015). We also expected to see negative health conditions and behaviors associated with those populations who have experienced IPV, consistent with prior literature (Basile et al., 2004; Black, 2011; Campbell, 2002; Cerulli et al., 2012; Creech et al., 2012; Smith et al., 2017; Wilson et al., 2007).

## Methods

New York City is an economically, racially, and ethnically diverse metropolis of ~ 8.2 million people. Our study population included noninstitutionalized NYC residents aged ≥ 18 years. The NYC CHS is a representative, annual, cross-sectional telephone survey that has been conducted annually since 2002, and serves as a public health surveillance tool for monitoring health conditions and behaviors (NYC DOHMH, 2020). CHS uses a stratified random sampling approach and in 2018 was weighted to the adult NYC residential population using the 2017 American Community Survey (ACS) (U.S. Census Bureau, 2017). The sampling frame is constructed using telephone numbers derived from a commercial vendor, including landlines and mobile telephones. Multiple attempts are made to reach someone at each number. Only one adult in each household is surveyed. The 2018 CHS had a cooperation rate of 82.8%, although the more conservative measure, response rate, was 8.4% (NYC DOHMH, 2020).

The survey asks approximately 125 questions concerning different topics and takes 25 min to complete (see supplementary material for survey questions used in this study). The survey is administered in English, Spanish, Russian, Chinese, Bengali, Haitian Creole, Hindi, Arabic, and Farsi. CHS was approved by the Institutional Review Boards (IRB) of the NYC DOHMH (#02–035) and Abt Associates (#0956). This analysis was approved by the NYC DOHMH IRB (#19–019) and was also reviewed by the Centers for Disease Control and Prevention (CDC) and conducted in a manner consistent with federal law and CDC policy.^1^

The exposure of ever having experienced psychological IPV was determined by asking “has a current or former intimate partner ever insulted you, or called you names repeatedly, or controlled your behavior?” The exposure of ever having experienced physical IPV was determined by asking “has a current or former intimate partner ever hit, slapped, shoved, choked, kicked, shaken, or otherwise physically hurt you?” Intimate partners were defined as “current or past boyfriends, girlfriends, husbands, wives, common-law spouses, dating partners, or someone with whom you have a child.”

The health conditions and behaviors that we examined were defined as follows: did not get needed mental health treatment (“Was there a time in the past 12 months when you needed treatment for a mental health problem but did not get it?”), did not get needed medical care (“Was there a time in the past 12 months when you needed medical care but did not get it? Medical care includes doctor’s visits, tests, procedures, prescription medication and hospitalizations”), current depression (determined as scoring 10–24 points on the Patient Health Questionnaire [PHQ-8]) (Kroenke et al., 2009), heavy drinking (defined as drinking > 2 alcoholic drinks per day for males or > 1 alcoholic drink per day for females, on average in the past 30 days), current smoking (two questions: “Have you smoked at least 100 cigarettes in your entire life?” and for those who responded yes, “Do you now smoke cigarettes every day, some days, or not at all?”), fair or poor health (“Would you say in general your health is excellent, very good, good, fair or poor?”), and hypertension (“Have you ever been told by a doctor, nurse or other health professional that you have hypertension, also called high blood pressure?”).

We examined the following sociodemographic variables: sex at birth, gender identity, race/ethnicity, marital status, age, sexual orientation, where born, neighborhood poverty, employment status and educational attainment. Gender identity response options were recoded as cis-gendered and transgendered/gender non-conforming. Race and ethnicity variables were analyzed such that Latinx includes people of Hispanic or Latin origin regardless of race. Black, White, and Asian and Pacific Islander racial categories exclude Latinx individuals. Multiracial individuals were categorized as ‘Other Race,’ but sample sizes were too small for analyses. Marital status response options were recoded as divorced, widowed, or separated; never married; and married or partnered. Employment status response options were recoded as employed, unemployed and not in the labor force. Education response options were recoded as less than high school, high school, some college, and college graduate. Neighborhood poverty is the percentage of residents in the zip code living below 100% of the federal poverty level as defined by the 2013–2017 ACS and categorized as low (0%– < 10%), medium (10%– < 20%), high (20%– < 30%), and very high (30%–100%) neighborhood poverty (U.S. Census Bureau, 2017).

Prevalence estimates were age-adjusted to the U.S. 2000 Standard Population, except for age-specific estimates (Klein & Schoenborn, 2001). To estimate prevalence, we conducted bivariate analyses, adjusted for age, and 95% CIs for psychological and physical IPV. Data were stratified by sex at birth, gender identity, race/ethnicity, marital status, age group, sexual orientation, place of birth (U.S.-born vs born outside the U.S.), neighborhood poverty level, and employment status. For respondents aged ≥ 25 years, data were stratified by educational attainment. Prevalence estimates were compared using paired *t*-tests with a designated reference category. We describe results where there are differences in patterns of association between overall and sex-stratified prevalence estimates. Estimates were identified as potentially unstable if the prevalence estimate had a relative standard error > 30% or if the sample size denominator was < 50. For both IPV types and for each health condition and behavior, we constructed directed acyclic graphs (DAGs) using CHS data and literature evidence to determine potential confounding variables (see supplementary material). While DAGs illustrate a causal framework, they also highlight the relationships between variables and can be used to identify a minimum set of confounding variables to create a parsimonious regression model (Moffa et al., 2017; Röhrig et al., 2014; Tennant et al., 2021). We performed a multivariable log-linear regression analysis for each health condition and behavior to estimate adjusted prevalence ratios (PRs) and 95% CIs, accounting for potential confounding. Both prevalence estimates and adjusted PRs were calculated with stratification by sex at birth to examine intersectionality of multiple identities. Models were grounded in theory and empirical literature evidence, and analyses had a priori hypotheses; as such, Bonferroni adjustments for multiple comparisons were not conducted. All analyses were conducted with SAS Enterprise Guide® 7.1 (SAS Institute, Inc., Cary, North Carolina) with SAS enabled SUDAAN® (RTI International, Research Triangle Park, North Carolina).

## Results

In 2018, the CHS had a total of 10,076 surveys that were completed. The results of the 2018 CHS are summarized in online materials including a codebook with the distribution of survey respondent demographics, and a disposition report of the outcome from all survey attempts (NYC DOHMH, 2020). Surveys with missing responses (131 [1.3%] for the psychological IPV question and 112 [1.1%] for the physical IPV question), were removed from analysis. The sample size used for analysis of psychological IPV was N = 9,945; and for the analysis of physical IPV was N = 9,964. Overall, 9.0% (95% CI: 8.1–9.9) of respondents reported experiencing both types of IPV. Results are presented as either physical or psychological IPV with respondents reporting both types of IPV included in both analyses.

### Psychological Intimate Partner Violence

A total of 16.7% (95% CI: 15.6–17.8) or an estimated 1,104,000 of NYC adults reported ever having experienced psychological IPV (Table 1). When comparing stratified variables to a reference using paired *t*-tests, we found that females (18.6%; 95% CI: 17.0–20.2) had a higher prevalence of psychological IPV than males (14.5%; 95% CI: 13.1–16.2, reference [ref]) (Table 1). Transgender and gender nonconforming adults had a psychological IPV prevalence of 27.3% (95% CI: 15.1–44.2). The prevalence of psychological IPV was similar among Latinx (17.7%; 95% CI: 15.8–19.9) and Black (18.1%; 95% CI: 15.7–20.8), and similar compared with White (18.1%; 95% CI: 16.1–20.3, ref) adults. However, those who identified as Asian or Pacific Islander (7.0%; 95% CI: 5.2–9.4) had a lower prevalence of psychological IPV than their White counterparts. Latino males (12.9%; 95% CI: 10.4–15.9) had a lower prevalence than White males (17.0%; 95% CI: 14.3–20.0, ref) (Table 2).

**Table 1:** Lifetime prevalence of psychological and physical intimate partner violence (IPV) by demographic variables—NYC Community Health Survey, 2018

Variable	Psychological IPV			Physical IPV		
	Prevalence (%)	95% CI	*P*-Value	Prevalence (%)	95% CI	*P*-Value
Total	16.7	15.6–17.8	–	9.8	9.0–10.7	–
Sex at birth						
Female Male	18.6 14.5	17.0–20.2 13.1–16.2	< 0.001 ref	12.4 6.8	11.1–13.8 5.8–8.0	< 0.001 ref
Gender identity						
Transgendered/gender nonconforming Cis-gendered	27.3^a^ 16.6	15.1–44.2 15.5–17.7	0.158 ref	18.7 ^a^ 9.7	10.1–32.0 8.9–10.6	0.108 ref
Race/ethnicity ^b^						
White Black Latinx Asian/Pacific Islander	18.1 18.1 17.7 7.0	16.1–20.3 15.7–20.8 15.8–19.9 5.2–9.4	ref 0.980 0.792 < 0.001	9.2 11.5 11.1 3.7	7.9–10.8 9.5–13.8 9.6–12.9 2.4–5.6	ref 0.094 0.090 < 0.001
Marital Status						
Divorced/widowed/separated Never married Married/partnered	28.1 19.3 13.0	22.5–34.4 17.2–21.6 11.3–14.8	< 0.001 < 0.001 ref	19.1 12.3 6.8	15.0–24.1 10.5–14.3 5.5–8.2	< 0.001 < 0.001 ref
Age Group (yrs)						
18–24 25–44 45–64 65 +	16.2 19.3 16.8 10.6	13.0–20.0 17.4–21.4 15.0–18.7 9.0–12.5	ref 0.125 0.756 0.006	6.7 11.9 10.3 6.3	4.7–9.4 10.4–13.6 8.9–11.9 5.0–7.9	ref < 0.001 0.010 0.788
Sexual Orientation						
Bisexual Lesbian or Gay Straight	29.5 25.6 16.1	22.0–38.3 18.6–34.1 15.0–17.3	0.002 0.019 ref	22.8 15.2 9.1	16.2–31.1 10.6–21.5 8.3–10.1	< 0.001 0.030 ref
U.S.-born vs born outside U.S						
U.S. Born Born Outside of U.S	21.2 11.8	19.6–23.0 10.4–13.2	ref < 0.001	12.8 6.6	11.5–14.2 5.7–7.8	ref < 0.001
Neighborhood Poverty						
Low (< 10%) Medium (10%– < 20%) High (20%– < 30%) Very High (30% +)	17.1 17.2 15.4 15.9	14.7–20.0 15.5–19.2 13.5–17.7 13.6–18.5	ref 0.949 0.325 0.506	7.5 10.1 10.8 9.7	5.9–9.5 8.8–11.7 9.1–12.6 7.8–11.9	ref 0.023 0.011 0.114
Employment status						
Employed Unemployed Not in labor force	17.0 20.9 18.4	15.5–18.6 16.6–25.8 16.0–21.2	ref 0.117 0.344	10.3 12.8 10.5	9.1–11.7 9.6–16.9 8.6–12.6	ref 0.200 0.908
Education^c^						
Less than High School High School Some College College Graduate	14.4 14.9 19.8 17.6	11.9–17.3 12.5–17.6 17.2–22.7 15.9–19.5	0.053 0.085 0.196 ref	9.2 8.6 13.4 10.4	7.3–11.7 6.9–10.8 11.2–16.0 9.1–12.0	0.361 0.150 0.036 ref

Data are age-adjusted to the U.S. 2000 Standard Population, except for age-specific estimates

Of 10,076 survey respondents, 9,945 answered the psychological IPV question, and 9,964 answered the physical IPV question

^a^ Estimate was identified as potentially unstable (e.g., the prevalence estimate had a relative standard error > 30% or the sample size denominator was < 50) and should be interpreted with caution

^b^ Latinx includes people of Hispanic or Latinx origin, regardless of race. Black, White, and Asian/Pacific Islander racial categories exclude Latinx

^c^ Education was restricted to individuals aged 25 years and older

**Table 2:** Lifetime prevalence of psychological intimate partner violence stratified by sex at birth and by demographic variables—NYC Community Health Survey, 2018

Variable	Female			Male		
	Prevalence (%)	95% CI	*P*-value	Prevalence (%)	95% CI	*P*-Value
Total	18.6	17.0–20.2	–	14.5	13.1–16.2	–
Race/ethnicity^a^						
White Black Latinx Asian/Pacific Islander	19.1 19.2 22.0 7.0	16.3–22.4 16.0–22.9 19.2–25.1 4.7–10.2	ref 0.98 0.181 < 0.001	17.0 16.7 12.9 7.1	14.3–20.0 13.3–20.8 10.4–15.9 4.5–11.0	ref 0.919 0.043 < 0.001
Marital Status						
Divorced/widowed/separated Never married Married/partnered	29.3 19.6 14.9	23.0–36.4 16.9–22.6 12.7–17.6	< 0.001 0.015 ref	30.0 20.4 10.6	22.5–38.8 16.9–24.3 8.6–13.1	< 0.001 < 0.001 ref
Age Group (yrs)						
18–24 25–44 45–64 65 +	16.6 21.7 19.1 11.7	12.0–22.5 19.0–24.7 16.7–21.9 9.6–14.3	ref 0.093 0.393 0.097	15.7 16.8 14.1 9.1	11.6–21.0 14.2–19.7 11.7–17.0 6.9–11.9	ref 0.702 0.560 0.013
Sexual Orientation						
Bisexual Lesbian or Gay Straight	34.6^b^ 20.5 17.7	24.9–45.8 12.7–31.5 16.1–19.4	0.002 0.564 ref	16.0^b^ 25.9 14.2	8.3–28.7 17.9–35.8 12.7–16.0	0.734 0.013 ref
U.S.-born vs born outside U.S						
U.S.-Born Born Outside of U.S	23.0 13.8	20.7–25.4 11.9–15.9	ref < 0.001	19.2 9.5	17.0–21.7 7.8–11.7	ref < 0.001
Neighborhood Poverty						
Low (< 10%) Medium (10%– < 20%) High (20%– < 30%) Very High (30% +)	16.1 19.1 18.7 19.0	13.0–19.9 16.6–21.8 15.8–22.1 15.8–22.8	ref 0.185 0.278 0.246	18.1 15.2 11.9 12.2	14.4–22.6 12.9–18.0 9.5–14.8 9.3–15.9	ref 0.236 0.012 0.027
Employment status						
Employed Unemployed Not in labor force	19.7 18.7 19.9	17.5–22.2 14.0–24.7 16.9–23.2	ref 0.745 0.938	14.6 23.3 14.7	12.7–16.7 16.4–31.9 10.9–19.5	ref 0.033 0.944
Education^c^						
Less than High School High School Some College College Graduate	19.0 14.6 23.3 19.5	15.2–23.5 11.6–18.3 19.6–27.5 17.1–22.1	0.862 0.023 0.108 ref	10.1 15.1 15.8 15.4	7.1–14.0 11.7–19.2 12.4–19.9 13.0–18.2	0.014 0.882 0.873 ref

Data are age-adjusted to the U.S. 2000 Standard Population, except for age-specific estimates

Of 10,076 survey respondents, 9,945 answered the psychological IPV question

^a^ Latinx includes people of Hispanic or Latin origin, regardless of race. Black, White, and Asian/Pacific Islander racial categories exclude Latinx

^b^ Estimate was identified as potentially unstable (e.g., the prevalence estimate had a relative standard error > 30% or the sample size denominator was < 50) and should be interpreted with caution

^c^ Education was restricted to individuals 25 years and older

As compared with prevalence among married or partnered adults (13.0%; 95% CI: 11.3–14.8, ref), prevalence was higher among adults who were divorced, separated or widowed (28.1%; 95% CI: 22.5–34.4) and among those who were never married (19.3%; 95% CI: 17.2–21.6). This pattern was also true overall and when stratified by sex at birth (Table 2). Prevalence by age group was similar for all age categories, but lower among adults aged ≥ 65 years (10.6%; 95% CI: 9.0–12.5), compared with adults aged 18–24 years (16.2%; 95% CI: 13.0–20.0, ref). Lesbian or gay (25.6%, 95% CI: 18.6–34.1) and bisexual adults (29.5%; 95% CI: 22.0–38.3) had a higher prevalence, compared with adults who identified as straight (16.1%, 95% CI: 15.0–17.3, ref). Prevalence was lower among adults born outside of the United States (11.8%; 95% CI: 10.4–13.2), compared with U.S.-born adults (21.2%; 95% CI: 19.6–23.0, ref). Overall, no differences were reported by neighborhood poverty, employment status, or education attainment (Table 1). However, when stratified by sex, unemployed males (23.3%; 95% CI: 16.4–31.9) had a higher prevalence than employed males (14.6%; 95% CI: 12.7–16.7, ref) (Table 2).

### Physical Intimate Partner Violence

A total of 9.8% (95% CI: 9.0–10.7) of NYC adults, or an estimated 650,000 people, reported ever having experienced physical IPV (Table 1). When comparing stratified variables to a reference using paired *t*-tests, we found that females (12.4%; 95% CI: 11.1–13.8) had a higher prevalence of physical IPV than males (6.8%; 95% CI: 5.8–8.0, ref) (Table 1). Transgender and gender nonconforming adults had a physical IPV prevalence of 18.7% (95% CI: 10.1–32.0). Reported prevalence of physical IPV was similar among Black (11.5%, 95% CI: 9.5–13.8) and Latinx adults (11.1%, 95% CI: 9.6–12.9), compared with White (9.2%, 95% CI: 7.9–10.8, ref) adults. However, Asian and Pacific Island-identified adults (3.7%, 95% CI: 2.4–5.6) had a lower prevalence of physical IPV, compared with White adults. Latina adults reported a higher prevalence (16.0%; 95% CI: 13.6–18.8), compared with White female adults (5.5%; 95% CI: 4.0–7.5, ref) (Table 3).

**Table 3:** Lifetime prevalence of physical intimate partner violence stratified by sex at birth and by demographic variables—NYC Community Health Survey, 2018

Variable	Female			Male		
	Prevalence (%)	95% CI	*P*-Value	Prevalence (%)	95% CI	*P*-Value
Total	12.4	11.1–13.8	–	6.8	5.8–8.0	–
Race/ethnicity^a^						
White Black Latinx Asian/Pacific Islander	10.4 13.6 16.0 4.0	8.4–12.8 10.8–17.1 13.6–18.8 2.3–6.8	ref 0.093 0.001 < 0.001	7.9 8.5 5.5 3.2^b^	6.2–10.1 6.1–11.7 4.0–7.5 1.7–6.1	ref 0.749 0.068 0.001
Marital Status						
Divorced/widowed/separated Never married Married/partnered	21.3 14.8 8.5	16.6–27.0 12.2–17.8 6.7–10.7	< 0.001 < 0.001 ref	21.6 10.1 4.6	14.7–30.6 7.7–13.2 3.4–6.1	< 0.001 < 0.001 ref
Age Group (yrs)						
18–24 25–44 45–64 65 +	7.4 14.9 13.2 8.6	4.5–11.7 12.6–17.6 11.1–15.6 6.6–11.0	ref 0.001 0.006 0.566	6.0 8.6 6.9 3.2	3.6–9.8 6.8–10.8 5.3–9.0 2.0–5.0	ref 0.163 0.605 0.098
Sexual Orientation						
Bisexual Lesbian or Gay Straight	30.8^b^ 17.5 11.4	20.4–43.5 9.7–29.3 10.1–12.9	0.001 0.228 ref	11.9^b^ 14.9 6.4	5.2–25.2 9.5–22.6 5.3–7.6	0.256 0.010 ref
U.S.-born vs born outside U.S						
U.S.-Born Born Outside of U.S	16.0 8.5	14.0–18.2 7.0–10.2	ref < 0.001	9.1 4.4	7.6–11.0 3.3–6.0	ref < 0.001
Neighborhood Poverty						
Low (< 10%) Medium (10%– < 20%) High (20%–30%) Very High (30% +)	7.2 13.1 14.9 11.6	5.3–9.8 11.0–15.5 12.3–18.0 9.1–14.8	ref < 0.001 < 0.001 0.017	7.8 6.7 6.1 7.0	5.4–11.1 5.2–8.6 4.5–8.1 4.7–10.4	ref 0.510 0.302 0.702
Employment status						
Employed Unemployed Not in labor force	13.5 14.2 12.3	11.5–15.8 10.1–19.6 10.0–15.0	ref 0.793 0.453	7.4 11.0 6.1	6.1–9.0 6.6–17.8 3.9–9.2	ref 0.213 0.373
Education^c^						
Less than High School High School Some College College Graduate	14.8 11.3 17.6 11.8	11.4–19.1 8.7–14.7 14.2–21.6 9.9–14.1	0.174 0.798 0.007 ref	3.1 5.7 8.6 8.9	1.8–5.3 3.6–8.8 6.2–11.8 7.1–11.1	< 0.001 0.046 0.843 ref

Data are age-adjusted to the U.S. 2000 Standard Population, except for age-specific estimates

Of 10,076 survey respondents, 9,964 answered the physical IPV question

^a^ Latinx includes people of Hispanic or Latin origin, regardless of race. Black, White, and Asian/Pacific Islander racial categories exclude Latinx

^b^ Estimate was identified as potentially unstable (e.g., the prevalence estimate had a relative standard error > 30% or the sample size denominator was < 50) and should be interpreted with caution

^c^ Education was restricted to individuals aged 25 years and older

As compared with adults who were married or partnered (6.8%; 95% CI: 5.5–8.2, ref), a higher prevalence of physical IPV was reported among adults who were divorced, separated, or widowed (19.1%; 95% CI: 15.0–24.1). This was also true for those who were never married (12.3%; 95% CI: 10.5–14.3). This pattern was also true overall and when stratified by sex. Overall, prevalence was higher among adults aged 25–44 years (11.9%; 95% CI: 10.4–13.6) and aged 45–64 years (10.3%; 95% CI: 8.9–11.9), compared with adults aged 18–24 years (6.7%; 95% CI: 4.7–9.4, ref) (Table 1). Similarly, when stratified by sex, females aged 25–44 years (14.9%; 95% CI: 12.6–17.6) and aged 45–64 years (13.2%; 95% CI: 11.1–15.6) had a higher prevalence than females aged 18–24 years (7.4%; 95% CI: 4.5–11.7, ref) (Table 3). A higher prevalence of physical IPV was reported among lesbian or gay (15.2%; 95% CI: 10.6–21.5) and bisexual (22.8%; 95% CI: 16.2–31.1) adults, compared with those who identified as straight (9.1%; 95% CI: 8.3–10.1, ref). Prevalence was lower among adults born outside of the United States (6.6%, 95% CI: 5.7–7.8), compared with U.S.-born adults (12.8%, 95% CI: 11.5–14.2, ref). Prevalence of physical IPV was higher among adults living in medium neighborhood poverty (10.1%; 95% CI: 8.8–11.7) or high neighborhood poverty (10.8%; 95% CI: 9.1–12.6), compared with low neighborhood poverty (7.5%, 95% CI: 5.9–9.5, ref). No differences by employment status were reported. Adults with some college education (13.4%; 95% CI: 11.2–16.0) had a higher prevalence of physical IPV, compared with adults who were college graduates (10.4%; 95% CI: 9.1–12.0, ref) (Table 1).

### Health Correlates of Psychological IPV

Adults who reported ever having experienced psychological IPV, compared with those who did not, had a higher prevalence of not getting needed treatment for a mental health problem (PR: 4.5; 95% CI: 3.3–6.1), current depression (PR: 2.6; 95% CI: 2.1–3.1), heavy drinking (PR: 2.1; 95% CI: 1.6–2.8), current smoking (PR: 1.7; 95% CI: 1.4–2.0), not getting needed medical care (PR: 1.7; 95% CI: 1.4–2.1), and fair or poor health (PR: 1.4; 95% CI: 1.2–1.6). Males who reported having experienced psychological IPV, compared with those who did not, had a higher prevalence of hypertension (PR: 1.4; 95% CI: 1.1–1.7), however, there was no difference among females who reporting having experienced psychological IPV compared with those who did not (PR: 1.1; 95% CI: 1.0–1.3) (Table 4).

**Table 4:** Adjusted prevalence ratios and 95% CIs for health conditions and behaviors among adults who reported ever experiencing psychological or physical intimate partner violence (IPV) —NYC Community Health Survey, 2018

	Psychological IPV			Physical IPV		
	Overall	Female	Male	Overall	Female	Male
Did not get needed mental health treatment^a^	4.5 (3.3–6.1)	4.8 (3.3–7.1)	3.8 (2.4–6.2)	3.9 (2.8–5.4)	3.7 (2.5–5.6)	3.8 (2.1–6.9)
Current depression^b^	2.6 (2.1–3.1)	3.0 (2.4–3.8)	2.0 (1.5–2.8)	2.6 (2.1–3.2)	2.8 (2.1–3.6)	2.2 (1.5–3.2)
Didn’t get needed medical care^c^	1.7 (1.4–2.1)	1.7 (1.3–2.2)	1.7 (1.2–2.3)	2.2 (1.8–2.7)	2.0 (1.5–2.7)	2.6 (1.8–3.7)
Heavy drinking^d^	2.1 (1.6–2.8)	1.8 (1.3–2.6)	2.6 (1.7–4.1)	1.9 (1.4–2.5)	1.8 (1.3–2.6)	2.0 (1.1–3.3)
Current smoking^e^	1.7 (1.4–2.0)	1.7 (1.4–2.2)	1.6 (1.3–2.1)	1.5 (1.2–1.8)	1.4 (1.1–1.9)	1.3 (1.0–1.8)
Fair or poor self-rated health^f^	1.4 (1.2–1.6)	1.4 (1.2–1.6)	1.5 (1.2–1.9)	1.2 (1.0–1.4)	1.2 (1.0–1.4)	1.3 (1.0–1.8)
Hypertension^g^	1.2 (1.1–1.4)	1.1 (1.0–1.3)	1.4 (1.1–1.7)	1.1 (0.9–1.3)	1.0 (0.8–1.2)	1.3 (1.0–1.7)

^a^ Psychological IPV model adjusted by sexual orientation; Physical IPV model adjusted by sexual orientation

^b^ Psychological IPV model adjusted by marital status, sex at birth, and sexual orientation; Physical IPV model adjusted by education, employment status, marital status, sex at birth, neighborhood poverty, and sexual orientation

^c^ Psychological IPV model adjusted by sex at birth, race/ethnicity, age group, and sexual orientation; Physical IPV adjusted by education, employment status, sex at birth, race/ethnicity, age group, and sexual orientation

^d^ Psychological IPV model adjusted by U.S. born, race/ethnicity, age group, and sexual orientation; Physical IPV model adjusted by education, employment status, U.S. born, race ethnicity, age group, and sexual orientation

^e^ Psychological IPV model adjusted by U.S. born, sex at birth, race/ethnicity, age group, sexual orientation, and marital status; Physical IPV model adjusted by education, marital status, U.S. born, sex at birth, race/ethnicity, age group, and sexual orientation

^f^ Psychological IPV model adjusted by marital status, U.S. born, sex at birth, race/ethnicity, age group, and sexual orientation; Physical IPV model adjusted by education, employment status, marital status, U.S. born, sex at birth, race/ethnicity, age group, neighborhood poverty, and sexual orientation

^g^ Psychological IPV model adjusted by marital status, race/ethnicity, age group, and sexual orientation; Physical IPV model adjusted by education, employment status, marital status, race/ethnicity, age group, neighborhood poverty, and sexual orientation

### Health Correlates of Physical IPV

Adults who reported ever having experienced physical IPV, compared with those who did not, had a higher prevalence of not getting needed mental health treatment (PR: 3.9; 95% CI: 2.8–5.4), current depression (PR: 2.6; 95% CI: 2.1–3.2), not getting needed medical care (PR: 2.2: 95% CI: 1.8–2.7), and heavy drinking (PR: 1.9; 95% CI: 1.4–2.5). Females who reported having experienced physical IPV, compared with those who did not, had a higher prevalence of current smoking (PR: 1.4; 95% CI: 1.1–1.9), but there was no difference in current smoking among males who reported physical IPV (PR: 1.3; 95% CI: 1.0–1.8). There was no difference in prevalence of having fair or poor self-rated health (PR: 1.2; 95% CI: 1.0–1.4) nor hypertension (PR: 1.1; 95% CI: 0.9–1.3) among adults who reported ever experiencing physical IPV compared with those who did not (Table 4).

## Discussion

We reported the lifetime age-adjusted prevalence of psychological and physical IPV among NYC adults. These data suggest that one in six (16.7%) NYC adults have experienced psychological IPV at some point in life, and one in 10 (9.8%) NYC adults have experienced physical IPV at some point in life. We found elevated prevalence of IPV among certain demographic groups. We report higher prevalence of both IPV types among females, as do other population-based surveys (Nybergh et al., 2013; Romans et al., 2007). The National Intimate Partner and Sexual Violence Survey found a higher prevalence of severe physical IPV among women, but no sex difference in the prevalence of being slapped, pushed, and shoved (Smith et al., 2018). However, the NYC CHS physical IPV exposure variable did not distinguish the level of violence severity. We found that the prevalence of experiencing physical IPV was higher in Latina adults than White female adults. This is consistent with the concept of intersectionality, where people with multiple identities who are historically marginalized experience disproportionate harms (Adams & Campbell, 2012). Gender, race, ethnicity, and other socioeconomic factors influence power between partners and propensity for experiencing IPV (Anderson, 1997; O’Neal & Beckman, 2017). Latina adults who have experienced IPV might face additional barriers when seeking social services including cultural, socioeconomic, and legal barriers (O’Neal & Beckman, 2017). Additionally, research suggests that transgender people might be at a particularly elevated risk of experiencing different forms of IPV (Yerke & DeFeo, 2016; West, 2012). In our study, point estimates of transgender and gender nonconforming people suggest higher prevalence of IPV, compared with cis-gendered individuals. However, our interpretation is limited because there were unstable prevalence estimates with wide confidence intervals due to small sample sizes.

Our data showed a higher lifetime prevalence of psychological and physical IPV among LGB adults, compared with adults who identify as straight, and these findings were consistent with existing literature (Walters et al., 2013; Edwards et al., 2015; Brown & Herman, 2015). Prior research suggests that discrimination and heterosexism experienced by LGB people might contribute to the elevated IPV prevalence within these communities (Edwards et al., 2015). Social services and research geared toward IPV in LGB communities are still new.

The higher prevalence of having ever experienced psychological and physical IPV found in our study among individuals who are divorced or never married, compared with those who are married or partnered in a couple, is also supported by prior literature evidence (Grande et al., 2003; Romans et al., 2007; Ruiz-Pérez et al., 2017; Vest et al., 2002). When IPV exists in a relationship, denial can be used as a coping strategy when an intimate partner causes harm and violence (Platt et al., 2009). For some people, the physical and emotional changes that can come with separation or divorce might enable someone to acknowledge their experiences as IPV. However, with these survey data, we cannot determine if IPV occurred before or after separation or divorce, which is a study limitation.

Considering that both IPV questions asked about lifetime exposure, it is unexpected that the age category of ≥ 65 years would have a lower prevalence for both psychological and physical IPV, because older individuals have more years of potential cumulative incidence (Warmling et al., 2017). Older adults might have experienced IPV at a time when abuse was viewed differently. The possibility exists that despite the focus on lifetime exposure, respondents might be forgetting or reframing prior life experiences or drawing from their recent memories. In addition, the impact of accumulated trauma might also affect memory as well (Streiner et al., 2009).

Our study found a lower prevalence of psychological and physical IPV in individuals born outside of the United States. Contrary to these findings, multiple studies have reported that immigrants are at a high risk of IPV (Ruiz Perez et al., 2017; Earner, 2010; Raj & Silverman, 2003), due in part to social isolation, socioeconomic factors, and level of acculturation (Raj & Silverman, 2003; Kim & Sung, 2016; Rai & Choi, 2018). Evidence suggests that IPV in immigrant communities might be underreported because of stigma, language barriers, immigration status, and fear of further victimization when trying to engage with provider services (Earner, 2010; Kim & Sung, 2016). Reduced reporting of IPV might be further exacerbated with increasing awareness of immigration enforcement (Muchow & Amuedo-Dorantes, 2020) and strengthening of anti-immigration policies (Huang, 2019).

Additionally, we found a lower prevalence of psychological and physical IPV among adults who identified as Asian or Pacific Islander, compared with White adults. Our prevalence estimates were lower than other literature findings; approximately 7% among Asian or Pacific Islander males and females as compared with 10–18% as described elsewhere (Chang et al., 2009). Many studies concerning IPV among Asian communities focus specifically on Asian immigrants and not U.S.-born Asian individuals (Ho et al., 2017, Rai & Choi, 2018). The racial category of Asian or Pacific Islander in our dataset includes both U.S.-born and immigrant individuals from dozens of different cultural backgrounds, each with a potentially distinct context and norm surrounding relationship behaviors and dynamics. Some research suggests that Asian cultures can place great emphasis on family cohesiveness and honor, and a potential explanation for low prevalence estimates could be underreporting for fear of disgracing the family (Wong et al., 2011). Culture, identity, and social position condition the understanding, the self-reporting, and experiences of IPV. When research findings depart from existing evidence, this highlights the sociocultural context and complexities of IPV, in addition to the respondent’s disclosure and readiness to seek help.

IPV is known to be associated with negative health behaviors and conditions (Basile et al., 2004; Black, 2011; Campbell, 2002; Cerulli et al., 2012; Creech et al., 2012; Smith et al., 2017; Wilson et al., 2007). However, the health conditions and behaviors correlated with different forms of IPV and magnitude of association have not previously been described in the context of a city such as NYC. In our study, the two correlates with the greatest measure of association for each IPV exposure were current depression and not getting needed mental health treatment. While it is biologically plausible that IPV could have a causal relationship with types of these health conditions and behaviors, the temporal relationship between IPV and these health conditions and behaviors cannot be established, and therefore our study cannot determine if IPV was responsible for causing them.

Associations between IPV and mental health symptoms are well supported in the literature. A meta-analysis of IPV and depression reported that 9%–28% of major depression disorders, elevated depressive symptoms, and postpartum depression were attributable to IPV (Beydoun et al., 2012). A focus group of IPV survivors, grounded in community-based participatory research principles, described that fear of the perpetrator played a pivotal role in prolonging psychological symptoms, even after the abuse had ended (Cerulli et al., 2012).

Barriers to getting mental health treatment for those who experience IPV include cost, lack of health insurance, psychological control by an abuser, low self-esteem, low self-efficacy, lack of knowledge about resources, and cultural considerations (Creech et al., 2012; Wilson et al., 2007). A 2002 study from the National Survey of Drug Use and Health reported that people who had experienced physical IPV were twice as likely to report unmet need for mental health treatment, even when accounting for socioeconomic factors (Lipsky & Caetano, 2007). A study on perinatal patients who received treatment at a psychiatric hospital, reported that more people than expected left treatment early if they were also experiencing psychological abuse (Creech et al., 2012).

Increasing access to quality care and improving mental health are both listed as high priorities for the City of New York and the NYC DOHMH (Mettey et al., 2015; NYC Mayor’s Office of ThriveNYC, 2018). Helping people to quit smoking, and drink responsibly are also stated DOHMH priorities and illustrated in these analyses as IPV behavior correlates (Mettey et al., 2015). The persistent and inescapable nature of IPV can influence many facets of health. Some researchers highlight the importance of collecting IPV data in health studies so that IPV can be further examined as a possible effect modifier or confounding variable of worse health outcomes for women (Sorenson & Saftlas, 1994).

We describe the benefits of using general population health surveys to investigate IPV epidemiology, however, we must consider these five limitations. Like many telephone-based surveys, CHS had a low response rate; statistical survey weighting was one approach used to abate possible nonresponse bias. While a low response rate alone does not indicate nonresponse bias, without analyzing non-respondents, we are unable to evaluate the contribution of potential nonresponse bias (Keeter et al., 2017; Lee et al., 2009). Secondly, we used a self-reported measure of IPV exposure. While there is very little evidence that IPV over-reporting occurs (Alhabib et al., 2010), many factors affect someone’s willingness to disclose prior IPV (Dienemann et al., 2005). This includes epidemiologic considerations such as survey design, number of questions, how questions are asked (Waltermaurer, 2005); if a respondent acknowledges their own experience as constituting a type of IPV; a respondent’s identity and intersectionality (Adams & Campbell, 2012); and safety considerations for respondents and their families (Dienemann et al., 2005). Self-disclosure in a survey might also be conditioned by systemic considerations, including trust in the institutions delivering the survey. Third, causality cannot be inferred with cross-sectional data, as the sequence in time cannot be established. Fourth, multiple IPV types such as physical and psychological IPV can co-occur in relationships and within a persons’ lifetime, yet the degree of this exposure overlap was not quantified in our study; this should be examined in future analyses. Lastly, for some populations our survey had small sample sizes; this occasionally resulted in unstable prevalence estimates and beyond sex at birth and one other socio-demographic variable, our ability to examine multiple intersecting strata was limited by sample size. Nonetheless, for health departments seeking to estimate IPV prevalence, understand associated health correlates, and identify levers for intervention, adding IPV-related questions to a general health survey can be informative, although limitations must be considered with interpretation.

The key implications of this analysis suggest that IPV potentially underlies public health priority health conditions and behaviors such as access to mental health treatment and depression among both male and female NYC residents. Understanding IPV prevalence and measuring its associated health and behavior comorbidities can aid healthcare and service providers in identifying which populations might be experiencing IPV to provide appropriate care and support. In addition, these data can help public health practitioners direct future interventions to those at elevated risk and can guide allocation of municipal resources to address and prevent these types of violence. At the time the authors write this paper, the coronavirus (COVID-19) pandemic has radically changed life for many. Public health crises, such as a pandemic, can cause grief, economic struggle, and physical and mental health challenges (Brooks et al., 2020). These physical and emotional challenges might be exacerbated during the pandemic for those who currently or previously have experienced IPV. Future IPV data collection efforts should consider IPV exposures potentially exacerbated by the COVID-19 pandemic. Attention, recognition, and measurement of IPV have important public health implications for resource allocation and prevention efforts.

## Supplementary Information

Below is the link to the electronic supplementary material.

Supplementary file1 (PDF 212 KB)

Supplementary file2 (PDF 226 KB)

Supplementary file3 (PDF 142 KB)

## Footnotes

1 See e.g., 45 C.F.R. part 46.102(l)(2), 21 C.F.R. part 56; 42 U.S.C. §241(d); 5 U.S.C. §552a; 44 U.S.C. §3501 et seq.
