# Supplementary material for: Psychological and Physical Intimate Partner Violence, Measured by the New York City Community Health Survey — New York City, 2018
Source: J Fam Violence. 2022 Sep 26:1–12. Online ahead of print. doi: 10.1007/s10896-022-00442-1 (PMC9510726; doi:10.1007/s10896-022-00442-1)
Supplement: Supplementary file 2 — Supplementary file2 (PDF 226 KB) [file 10896_2022_442_MOESM2_ESM.pdf]

Directed acyclic graphs for adjusted prevalence ratio models of physical intimate partner violence

Did not get needed mental health treatment

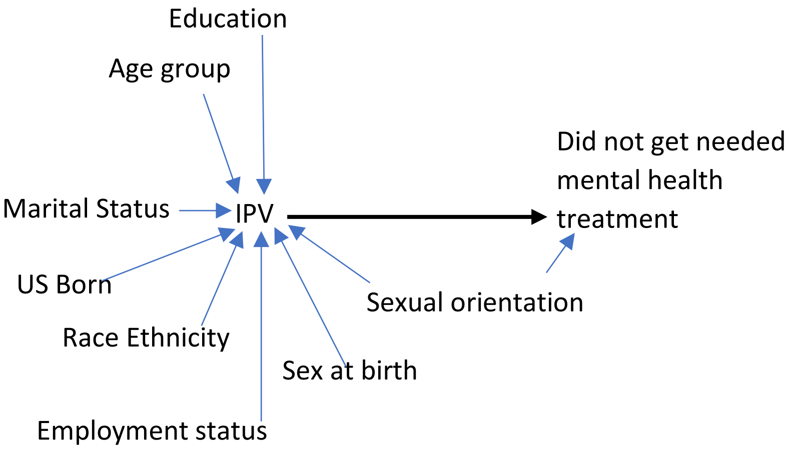

Data supporting DAG relationships:

- NYC Community Health Survey, 2016
- Haney, J. L. (2020). Sexual orientation, social determinants of health, and unmet substance use treatment need: Findings from a national survey. *Substance Use & Misuse*, *56*(2), 205-213.

Didn't get needed medical care

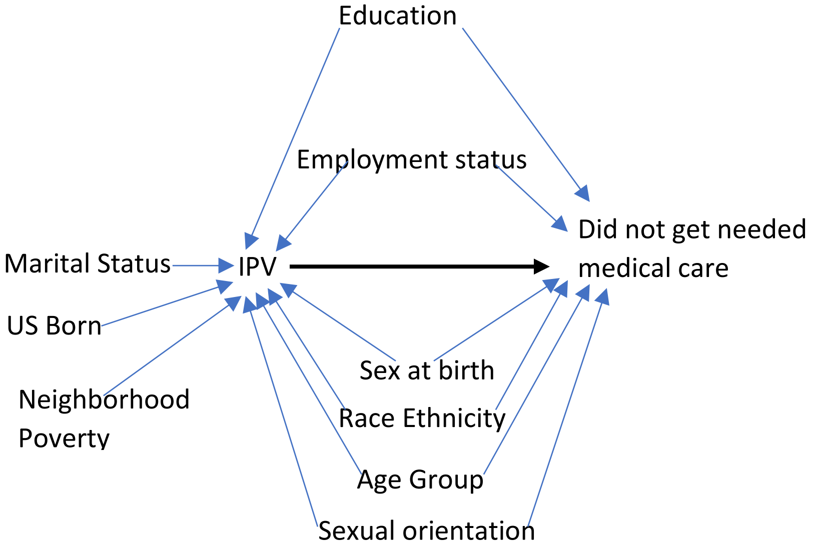

Data supporting DAG relationships:

- NYC Community Health Survey, 2016 and 2017
- Conron, K. J., Mimiaga, M. J., & Landers, S. J. (2010). A population-based study of sexual orientation identity and gender differences in adult health. *American Journal of Public Health*, *100*(10), 1953-1960.

Directed acyclic graphs for adjusted prevalence ratio models of physical intimate partner violence

Heavy drinking

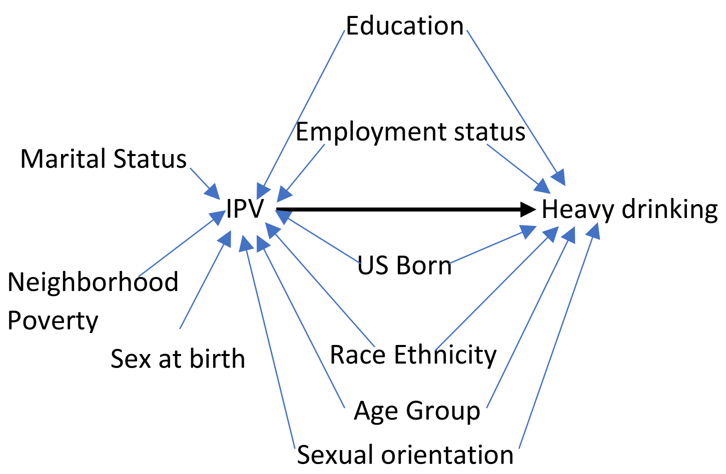

Data supporting DAG relationships:

- NYC Community Health Survey, 2016 and 2017
- Jackson, C. L., Agénor, M., Johnson, D. A., Austin, S. B., & Kawachi, I. (2016). Sexual orientation identity disparities in health behaviors, outcomes, and services use among men and women in the United States: a cross-sectional study. *BMC Public Health*, *16*(1), 1-11.

Current smoking

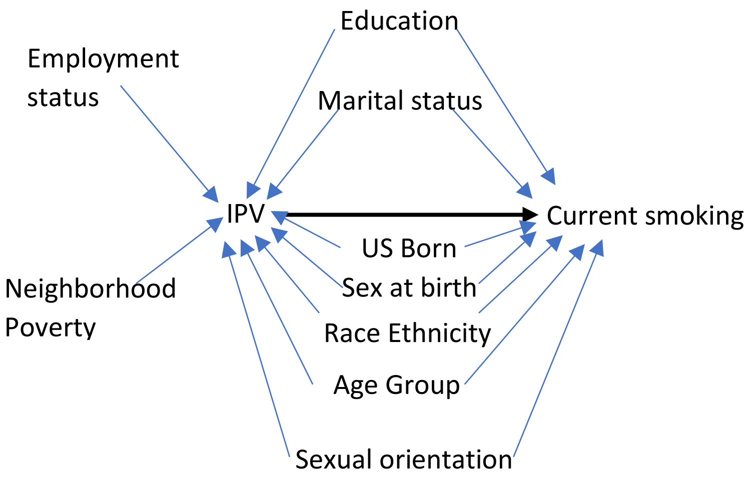

Data supporting DAG relationships:

- NYC Community Health Survey, 2016 and 2017
- Jackson, C. L., Agénor, M., Johnson, D. A., Austin, S. B., & Kawachi, I. (2016). Sexual orientation identity disparities in health behaviors, outcomes, and services use among men and women in the United States: a cross-sectional study. *BMC Public Health*, *16*(1), 1-11.

Directed acyclic graphs for adjusted prevalence ratio models of physical intimate partner violence

Current depression

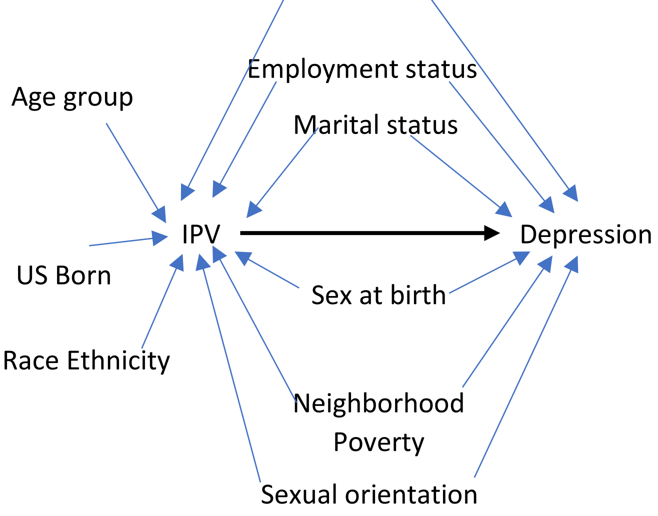

Data supporting DAG relationships:

- NYC Community Health Survey, 2016 and 2017
- Hatzenbuehler, M. L., Hilt, L. M., & Nolen-Hoeksema, S. (2010). Gender, sexual orientation, and vulnerability to depression. In *Handbook of Gender Research in Psychology* (pp. 133-151). Springer, New York, NY.

Fair or poor self-rated health

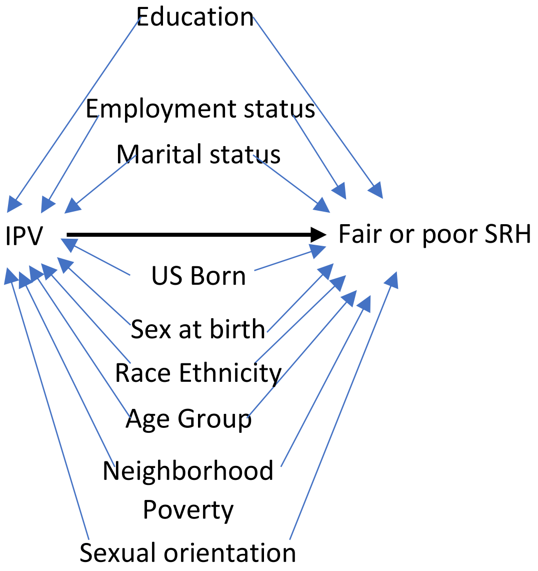

Data supporting DAG relationships:

- NYC Community Health Survey, 2016 and 2017
- Veenstra, G. (2011). Race, gender, class, and sexual orientation: intersecting axes of inequality and self-rated health in Canada. *International Journal for Equity in Health*, *10*(1), 1-11.

Directed acyclic graphs for adjusted prevalence ratio models of physical intimate partner violence

Hypertension

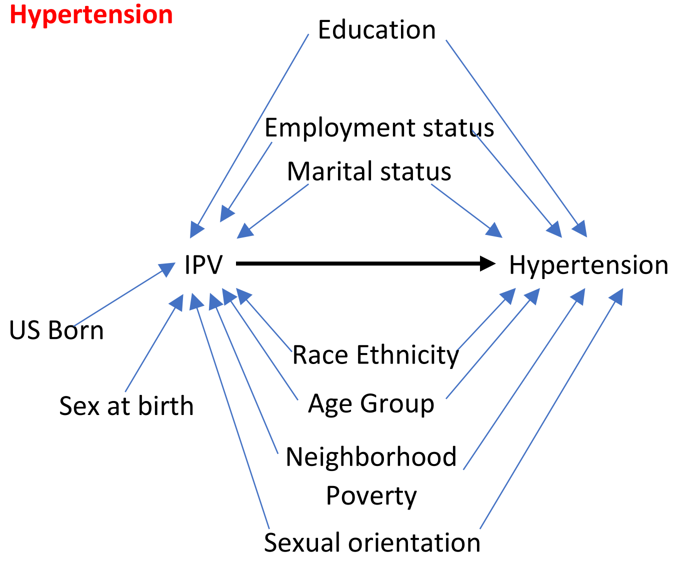

Data supporting DAG relationships:

- NYC Community Health Survey, 2016 and 2017
- Everett, B., & Mollborn, S. (2013). Differences in hypertension by sexual orientation among US young adults. *Journal of Community Health*, *38*(3), 588-596.
