# Supplementary material for: Psychological and Physical Intimate Partner Violence, Measured by the New York City Community Health Survey — New York City, 2018
Source: J Fam Violence. 2022 Sep 26:1–12. Online ahead of print. doi: 10.1007/s10896-022-00442-1 (PMC9510726; doi:10.1007/s10896-022-00442-1)
Supplement: Supplementary file 3 — Supplementary file3 (PDF 142 KB) [file 10896_2022_442_MOESM3_ESM.pdf]

NYC Community Health Survey Questions to estimate intimate partner violence, 2018. The complete 2018 survey instrument, including question order and interviewer notes, is available online at the Health Department website (https://www1.nyc.gov/site/doh/data/data-sets/community-health-survey-public-use-data.page).

Overall	Question wording
Physical IPV	**Ask all**<br>Has a current or former intimate partner ever hit, slapped, shoved, choked, kicked, shaken, or otherwise physically hurt you?<br>•Yes<br>• No
Psychological IPV	**Ask all**<br>Has a current or former intimate partner ever insulted you, or called you names repeatedly, or controlled your behavior?<br>•Yes<br>• No
**Demographic Variables**	**Question wording**
Sex at birth	**Ask all**<br>What was your sex assigned at birth? Male or female?<br>• Male<br>• Female
Gender identity	**Ask all**<br>How do you describe yourself?<br>• Cisgender Man<br>• Cisgender Woman<br>• Transgender Man<br>• Transgender Woman<br>• Gender non-conforming Man<br>• Gender non-conforming Woman
Ethnicity	**Ask all**<br>Are you Hispanic or Latino/Latina?<br>•Yes<br>• No
Race	**Ask all**<br>Which one or more of the following would you use to describe yourself? Would you describe yourself as….<br>• White<br>• Black or Black American<br>• Asian<br>• Middle Eastern or North African<br>• Native Hawaiian or Other Pacific Islander<br>• American Indian, Native, First Nations, Indigenous Peoples of the Americas, or Alaska Native, or<br>• Something else? (SPECIFY: How do you describe yourself?) ___________<br>• Hispanic/Latino

Marital Status	**Ask all**<br>Are you …<br>• Married<br>• Divorced<br>• Widowed<br>• Separated<br>• Never married<br>• a member of an unmarried couple living together or partnered
Age	**Ask all**<br>What is your age?
**Demographics continued**	**Question wording**
Sexual Orientation	**Ask all**<br>Are you …<br>• Gay or lesbian<br>• Straight, that is not gay<br>• Bisexual<br>• Something else
Where born - foreign/US	**Ask all**<br>Where were you born? Please tell me the country or US territory.<br>• USA<br>• Outside USA
Neighborhood poverty	**Ask all**<br>Could I please have your five-digit zip code? [Or: Just to confirm is your zip code _____?]
Employment	**Ask all**<br>Are you currently. . . .<br>• Employed for wages or salary<br>• Self-employed<br>• A Homemaker<br>• A Student<br>• Retired<br>• Unable to work<br>• Unemployed for 1 year or more<br>• Unemployed for less than 1 year

Educational attainment	**Ask all**<br>What is the highest grade or year of school you completed?<br>• Never attended school or only attended kindergarten<br>• Grades 1 through 8 (elementary)<br>• Grades 9 through 11 (some high school)<br>• Grade 12 or GED (high school graduate)<br>• College 1 year to 3 years (some college or technical school)<br>• College 4 years or more (college graduate)
**Health Conditions and Behaviors**	**Question wording**
Did not get needed mental health treatment	**Ask all**<br>Was there a time in the past 12 months when you needed treatment for a mental health problem but did not get it?<br>• Yes<br>• No
Did not get needed medical care	**Ask all**<br>Was there a time in the past 12 months when you needed medical care but did not get it? Medical care includes doctor's visits, tests, procedures, prescription medication and hospitalizations<br>• Yes<br>• No
Heavy drinking	**Ask all**<br>A drink of alcohol is 1 can or bottle of beer, 1 glass of wine, 1 can or bottle of wine cooler, 1 cocktail, or 1 shot of liquor. During the past 30 days, how many days per week or per month did you have at least 1 drink of any alcoholic beverage?<br>• Yes<br>• No
Current smoking	**Ask all**<br>Have you smoked at least 100 cigarettes in your entire life?<br>• Yes<br>• No
	**If yes, ask**<br>Do you now smoke cigarettes every day, some days, or not at all?<br>• Every day<br>• Some days<br>• Not at all
Fair or poor health	**Ask all**<br>Would you say in general your health is excellent, very good, good, fair or poor?<br>• Excellent<br>• Very good<br>• Good<br>• Fair<br>• Poor

Hypertension	**Ask all**<br>Have you ever been told by a doctor, nurse or other health professional that you have hypertension, also called high blood pressure?<br>• Yes<br>• No
The remaining 8 questions represent the Patient Health Questionnaire (PHQ)-8, an eight item screening instrument that assesses the frequency of depression symptoms over the past 2 weeks.6 A score of 10 to 24 points, indicative of moderate to severe depressive symptoms, was defined as current depression.	
Little interest	**Ask all**<br>Over the last 2 weeks, how often have you been bothered by: little interest or pleasure in doing things?<br>• Not at all<br>• Several days<br>• More than half the days<br>• Nearly every day
Down, depressed, or hopeless	**Ask all**<br>Over the last 2 weeks, how often have you been bothered by: feeling down, depressed or hopeless?<br>• Not at all<br>• Several days<br>• More than half the days<br>• Nearly every day
Trouble falling asleep, staying asleep, or sleeping too much	**Ask all**<br>Over the last 2 weeks, how often have you been bothered by: trouble falling or staying asleep, or sleeping too much?<br>• Not at all<br>• Several days<br>• More than half the days<br>• Nearly every day
Feeling tired, having little energy	**Ask all**<br>Over the last 2 weeks, how often have you been bothered by: feeling tired or having little energy?<br>• Not at all<br>• Several days<br>• More than half the days<br>• Nearly every day
Poor appetite or overeating	**Ask all**<br>Over the last 2 weeks, how often have you been bothered by: poor appetite or overeating?<br>• Not at all<br>• Several days<br>• More than half the days<br>• Nearly every day
Feeling bad about yourself	**Ask all**<br>Over the last 2 weeks, how often have you been bothered by: feeling bad about yourself – or that you are a failure or have let yourself or your family down?

	• Not at all<br>• Several days<br>• More than half the days<br>• Nearly every day
Trouble concentrating	**Ask all**<br>Over the last 2 weeks, how often have you been bothered by: trouble concentrating on things, such as reading the newspaper or watching TV?<br>• Not at all<br>• Several days<br>• More than half the days<br>• Nearly every day
